# Comparison of salvage therapies for isolated para-aortic lymph node recurrence in patients with uterine cervical cancer after definitive treatment

**DOI:** 10.1186/s13014-019-1442-6

**Published:** 2019-12-26

**Authors:** Hikaru Kubota, Kayoko Tsujino, Nor Shazrina Sulaiman, Shuhei Sekii, Yoko Matsumoto, Yosuke Ota, Toshinori Soejima, Satoshi Yamaguchi, Ryohei Sasaki

**Affiliations:** 1 Department of Radiation Oncology, Kobe University Hospital, 7-5-2 Kusunoki-cho, Chuo-ku, Kobe, Hyogo Japan; 2 Department of Radiation Oncology, Hyogo Cancer Center, 13-70, Kita-oji, Akashi, Hyogo Japan; 3 Department of Radiation Oncology, Kobe Proton Center, 1-6-8, Minatojima-minami-cho, Chuo-ku, Kobe, Japan; 4 Department of Gynecologic Oncology, Hyogo Cancer Center, 13-70, Kita-oji, Akashi, Hyogo Japan

**Keywords:** Uterine cervical cancer, Isolated Para-aortic lymph node recurrence, Chemoradiation therapy, Oligometastasis

## Abstract

**Background:**

Some studies have demonstrated that concurrent chemo-radiotherapy is an effective salvage treatment for isolated para-aortic lymph node (PALN) recurrence.

However, no studies have compared multi-treatment modalities, such as radiation therapy (RT), concurrent chemoradiotherapy (CCRT), surgery, chemotherapy, and best supportive care (BSC), across a sufficient number of patients with PALN recurrence. We thus aimed to evaluate the clinical outcomes of multi-treatment modalities for isolated PALN recurrence in uterine cervical cancer.

**Methods:**

Records of 50 patients who were first diagnosed with isolated PALN recurrence after definitive cervical cancer treatment from 2002 to 2016 at our institution were reviewed retrospectively. The initial definitive cervical cancer therapies included RT alone, CCRT, or surgery with or without post-operative RT. The median follow-up time was 33 months. The median age at recurrence diagnosis was 57 years (range, 26–84 years). The median duration between the end of initial treatment and recurrence was 10 months (range, 1–91 months). The median maximum metastatic lesion size was 17 mm (range, 8–60 mm). Twenty-four patients had one or two PALN metastases, while 26 had 3 or more. Eighteen patients were treated for recurrence with RT alone, seven with CCRT, three with surgery, 17 with chemotherapy, and five with BSC. Potential prognostic factors included histopathology, initial FIGO stage, initial treatment, age at recurrence, tumor markers (serum SCC-Ag and CEA) at recurrence, time to recurrence, maximum size of the metastatic lesion, number of metastases, and the recurrence treatment method.

**Results:**

The 3-year overall survival (OS) rates of all patients were 47.0%. The 3-year OS rate of patients who underwent CCRT for recurrence was 85.7%; surgery, 66.7%; chemotherapy, 48.8%; RT, 41.3%; and BSC, 0% (*p* = 0.014). Univariate analysis revealed that only the recurrence treatment method was significantly associated with OS. The 3-year local control rate (LCR) and progression free survival (PFS) rate for CCRT were 100 and 71.4%; for surgery, 100 and 66.7%; for chemotherapy, 33.6 and 13.7%; and for RT, 55.5 and 14.1%, respectively (LCR: *p* = 0.028, PFS: *p* = 0.059). The number of metastatic lesions, SCC-Ag levels and recurrence treatment method were significantly associated with LCR. Age at recurrence, SCC-Ag levels, and number of metastatic lesions were significantly associated with PFS.

**Conclusions:**

Although our patient cohort size was small, our results suggest that CCRT may be effective in preventing local disease recurrence in the PALN and may improve OS.

## Background

Uterine cervical cancer is one of the most common gynecologic cancers in Japan and has been associated with an excellent tumor control rate and favorable prognosis after definitive radiotherapy (RT), radical hysterectomy with pelvic lymph node dissection for early stages, and chemo-radiation therapy (CCRT) for locally advanced stages. However, approximately one-third of patients who are treated for cervical cancer develop recurrence [1, 2]. The most common recurrence sites, other than the pelvis, are the para-aortic lymph nodes [1]. Recurrences of uterine cervical cancer in isolated para-aortic lymph nodes (PALNs) after definitive treatment occur in 1.7 to 12% of patients [3–7]. Although PALN recurrences are defined as distant metastases, some patients with PALN recurrences have survived for long periods and were considered to be cured as nodal oligo-recurrence. Some reports [5–7] have shown that concurrent chemo-radiotherapy is an effective salvage treatment for isolated PALN recurrence.

However, no studies have compared multi-treatment modalities, such as radiation therapy (RT), concurrent chemoradiotherapy (CCRT), surgery, chemotherapy, and best supportive care (BSC), across a sufficient number of patients with PALN recurrences. The aim of this study was to evaluate the outcome of patients with isolated PALN recurrence after definitive treatment for primary cervical cancer at our institution.

## Methods

This study retrospectively examined the data of 50 patients who were first diagnosed with isolated PALN metastases after definitive cervical cancer treatment, from 2002 to 2016, at our institution. Initial definitive treatments were performed with RT alone, CCRT, or surgery with or without post-operative RT (PORT). The median follow-up time was 33 months, and the baseline patient characteristics are summarized in Table 1. The median age was 57 years (range, 26–84 years), the median time to PALN relapse was 10 months (range, 1–91 months), and the median maximum metastatic lesion size was 17 mm (range, 8–60 mm). Forty patients presented with squamous cell carcinoma and 10 with other histopathologies. Eleven patients had initial international federation of Gynecology and Obstetrics (FIGO) stage I disease, 24 had stage II, 10 had stage III, and five had stage IV disease. For PALN recurrence, 18 (36%) patients were treated with RT alone, 17 (34%) with chemotherapy, seven (14%) with CCRT, five (10%) with BSC, and three (6%) with surgery. The patient characteristics of each treatment group are summarized in Additional file 1: Table S1. There were no significant differences between the treatment groups in terms of the assessed variables except age at recurrence. PALN recurrence was defined as the appearance of clinical and radiologic evidence of disease. Radiologically, enlarged PALNs over 1 cm in size on a follow-up computed tomography (CT) scan or/and abnormal uptake on positron emission tomography with [18F]-fluorodeoxyglucose (FDG-PET) scans were defined as recurrences. Twenty-seven patients were diagnosed with FDG-PET scans. The maximum size of the metastatic lesion or the number of metastases were measured and evaluated by CT or FDG-PET CT. Based on the number of metastases, the cases were classified into two groups: 1–2 or ≥ 3. Two tumor markers, serum squamous cell carcinoma antigen (SCC-Ag) and carcinoembryonic antigen (CEA) were measured at the time of PALN recurrence. Elevated serum SCC-Ag (≥1.5 ng/mL) and CEA (≥5.0 ng/mL) levels were considered positive, whereas lower levels were considered negative.

**Table 1:** Patient characteristics

Variable		No.	%
Histopathology	Squamous cell carcinoma	40	80
	Adenocarcinoma	7	14
	Adenosquamous carcinoma	1	2
	Neuroendcrine carcinoma	1	2
	Carcinoma with rhabdoid feature	1	2
Initial FIGO stage	I	11	22
	II	24	48
	III	10	20
	IV	5	10
Initial treatment	Surgery with PORT	23	46
	Surgery without PORT	5	10
	CCRT	17	34
	RT alone	5	10
Age at the recurrence (year)	Median (range)	57 (26–84)	
Serum SCC at the recurrence	Positive	26	43
	Negative	17	28
	Missing	18	30
Serum CEA at the recurrence	Positive	26	43
	Negative	17	28
	Missing	18	30
Time interval between completion of initial treatment and PALN recurrence (month)	Median (range)	10 (1–91)	
Maximum size of PALN recurrence (mm)	Median (range)	17 (8–60)	
Number of recurrent PALN	1–2	24	48
	≥ 3	26	52
Treatment for PALN recurrence	RT alone	18	36
	CCRT	7	14
	Surgery	3	6
	Chemotherapy	17	34
	BSC	5	10

*Abbreviations: *FIGO* International Federation of Gynecology and Obstetrics, *Port* Post-operative radiation therapy, *serum SCC* Serum squamous cell carcinoma antigen, *serum CEA* Serum carcinoembryonic antigen, *CCRT* Concurrent chemoradiation therapy, *RT* Radiation therapy, *PALN* Para-aortic lymph nodes, *BSC* Best supportive care

Patients who had any evidence of metastatic disease in addition to PALN recurrence were excluded from this analysis.

The Institutional Review Board (IRB) of our institution approved this study (R-514).

The median follow-up time was 33 months, and the baseline patient characteristics are summarized in Table 1. The median age was 57 years (range, 26–84 years), the median time to PALN relapse was 10 months (range, 1–91 months), and the median maximum metastatic lesion size was 17 mm (range, 8–60 mm). For PALN recurrence, 18 (36%) patients were treated with RT alone, 17 (34%) with chemotherapy, seven (14%) with CCRT, five (10%) with BSC, and three (6%) with surgery.

### RT or CCRT

RT was performed with 10-MV photons using linear accelerators. A three-dimensional treatment-planning system was used for all patients, and all patients underwent four fields of irradiation. The RT target volume was the regional PALN area followed by PALN metastases, except in one patient in whom the target volume was the regional pelvic and PALN areas. For the RT field of prophylactic PALN irradiation, the superior margin was at the T12-L1 intervertebral space, and the inferior margin was located between the L4 and L5 intervertebral space or matched to the superior edge of the previous pelvic port. In boost irradiation for PALN metastases, the 5-mm margin to PALN metastases was added to the clinical target volume (CTV). A 5-mm setup margin for clinical target volume (CTV) was added to the planned target volume (PTV). Irradiation fields were obtained by providing a 5 mm leaf margin for the PTV. Doses were prescribed to the isocenter of each field. The median dose for the regional PALN area was 44 Gy (range; 39.6–50 Gy). The median dose for PALN metastases was 55 Gy (range: 50–58 Gy). All patients were treated using 2.0 Gy per fraction or 1.8 Gy per fraction. Concurrent chemotherapy was performed with weekly cisplatin 40 mg/m^2^ or weekly nedaplatin 30 mg/m^2^.

### Chemotherapy

Of the 17 patients who received chemotherapy, the chemotherapy regimens were paclitaxel/carboplatin in seven, docetaxel/carboplatin in four, irinotecan/cisplatin in three, irinotecan/nedaplatin in one, paclitaxel alone in one, and docetaxel alone in one.

### Surgery

Of the three patients who received surgery, para-aortic lymph node dissections were performed in two, and metastatic lymph node resection was performed in one. There was no operative mortality. Postoperative chemotherapy was performed in two patients, with paclitaxel/carboplatin in one and irinotecan in the other.

### Potential prognostic factors

Prognostic factors were evaluated for their impact on overall survival (OS), local control rate (LCR), and progression-free survival (PFS). Potential prognostic factors included histopathology, initial FIGO stage, initial treatment, age at recurrence, tumor markers (serum SCC-Ag and CEA) at recurrence, time to recurrence, maximum size of the metastatic lesion, number of metastases, and the recurrence treatment method.

### Toxicity

Toxicities were evaluated according to the Common Terminology Criteria for Adverse Events (CTCAE) v4.0. In the RT alone and CCRT arms, acute toxicity was defined as toxicity that occurred within 90 days after completion of radiation therapy, and any toxicity that occurred more than 90 days after treatment was regarded as late toxicity.

### Follow-up and statistical analyses

Physical examination, laboratory tests to evaluate serum tumor markers, SCC and/or CEA, chest-abdominal CT scans, and FDG-PET scans were used for the follow-up analysis. After completion of the treatment, patients were reviewed within 4–6 weeks, then every 3 months in the first 2 years, and every 4 months thereafter. Patients were evaluated with CT scans 1 month after treatment completion, and every 3–6 months thereafter. During follow-up, if the tumor marker levels elevated or if examination findings suggested possible recurrence, we performed CT scans or FDG-PET scans earlier than planned.

The database was analyzed using a software program (SPSS, version 24; IBM, Armonk, NY). OS, LCR, and PFS were evaluated using the Kaplan Meier method, and prognostic factors were evaluated using the log-rank test. Survival time and LCR were calculated from the diagnosis of PALN recurrence until death or a censored date. Statistical significance was defined as *p*-values of 0.05 or less, based on two-sided tests. Missing data were excluded from the analysis.

## Results

**Fig. 1:**
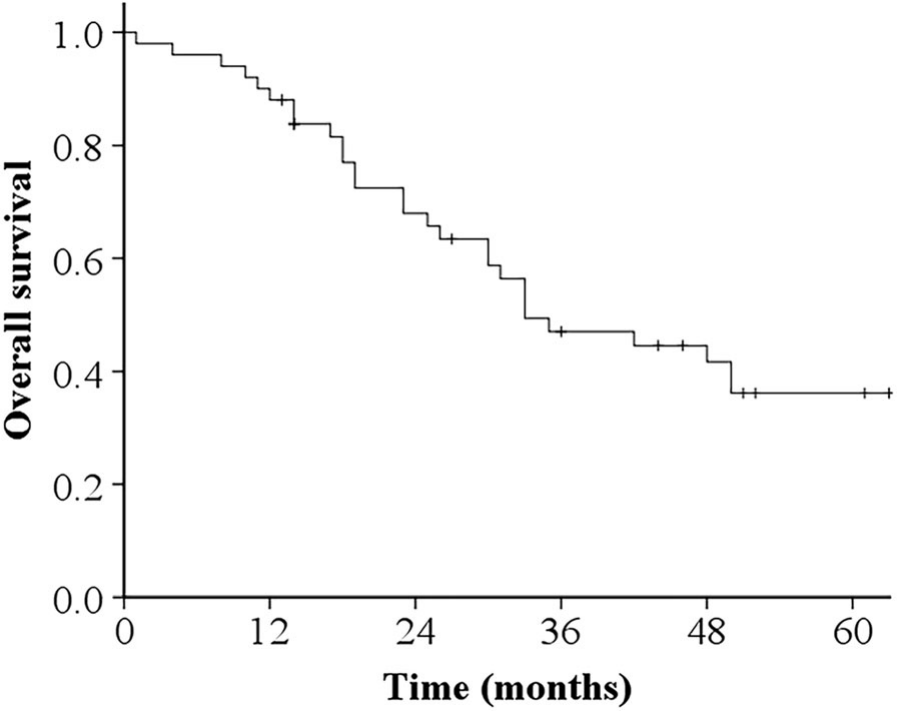
Overall survival (OS) curve of all patients. The 3- and 5-year OS rates were 47.0 and 36.2%, respectively

**Fig. 2:**
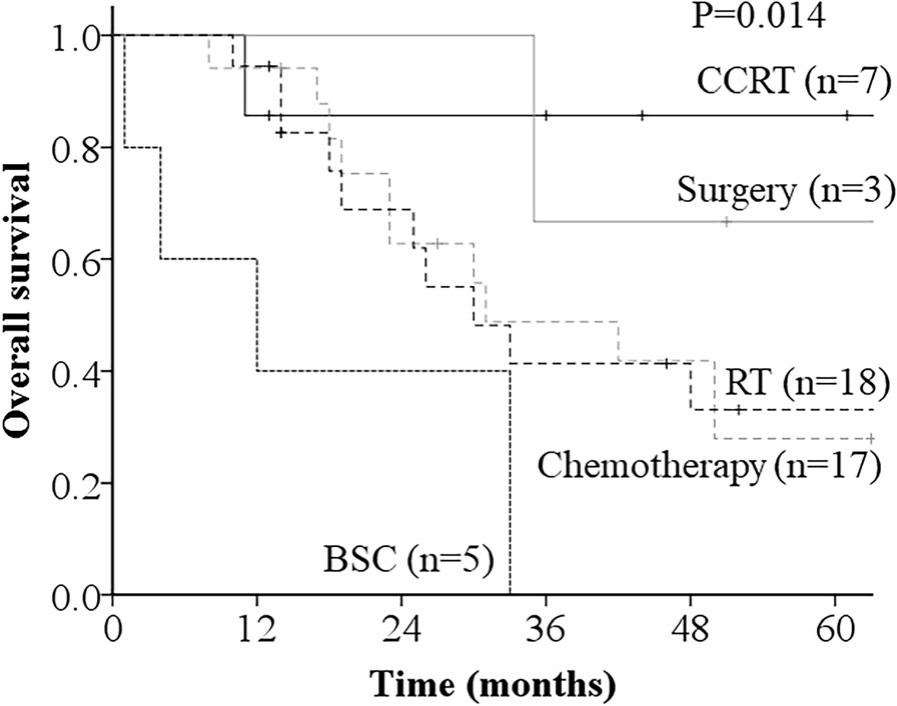
Overall survival (OS) curves according to the recurrence treatment method. Patients treated with concurrent chemoradiation therapy (CCRT) presented significantly superior OS than other patients (*p* = 0.014)

The 3-year OS rates of all patients were 47.0 and 36.2%, respectively (Fig. 1). With respect to the 3-year OS according to the PALN recurrence treatment method, the 3-year OS of patients who underwent CCRT was 85.7%, surgery, 66.7%; chemotherapy, 48.8%; RT, 41.3%; and BSC, 0% (*p* = 0.014) (Fig. 2). A clear significant correlation was observed between the PALN recurrence treatment method and overall survival. Of all the potential prognostic factors that we investigated, only the recurrence treatment method was significantly associated with OS in the univariate analysis (Table 2).

**Table 2:** Univariate analysis for OS, LCR and PFS

	n	OS			LCR			PFS		
		MST (months)	3-year (%)	p	MST (months)	3-year (%)	p	MST (months)	3-year (%)	p
Histopathology										
SCC	40	35	48.9		–	58.4		10	31.3	
The others	10	25	40	0.351	16	46.9	0.446	14	25	0.849
Initial FIGO										
I	11	33	37.9		–	25		8	9.1	
II	24	48	56.9		–	62.9		13	38.6	
III	10	35	42		–	63.5		13	20.8	
IV	5	33	26.7	0.481	–	100	0.184	–	66.7	0.24
Initial treatment										
Surgery with PORT	23	33	44.1		13	38.2		8	13.6	
Surgery without PORT	5	–	60		–	75		–	60	
CCRT	17	35	47.9		–	80.8		15	40.7	
RT alone	5	12	40	0.383	9	50	0.131	9	50	0.154
Age at the recurrence										
> 57	25	48	59.4		–	68.8		20	43.6	
≦57	25	25	35.3	0.152	17	45.1	0.14	7	18.3	0.031
Serum SCC-Ag at the recurrence										
Positive	26	31	38.8		13	37.2		7	13.3	
Negative	17	50	61.1	0.158	–	86.7	0.012	–	53.6	0.002
Serum CEA at the recurrence										
Positive	17	30	41.2		13	40.2		7	12.5	
Negative	26	48	51.9	0.227	–	66.1	0.081	17	45	0.070
Time to recurrence										
> 10	24	48	57.6		–	61.1		14	34.6	
≦10	26	33	38.1	0.498	–	51.8	0.404	9	24.1	0.281
Maximum size of PALN recurrence										
> 17 mm	21	23	38.3		13	46.6		5	25.3	
≦17 mm	29	42	53	0.587	–	62.6	0.237	15	33.2	0.078
Number of PALN recurrence										
1–2	24	48	55.3		9	82		16	44.5	
3 and more	26	30	38.8	0.244	–	36.1	0.001	7	16.5	0.009
Treatment for PALN recurrence										
RT alone	18	30	41.3		–	55.5		9	14.1	
CCRT	7	–	85.7		–	100		–	71.4	
Surgery	3	–	66.7		–	100		–	66.7	
Chemotherapy	17	31	48.8		–	33.6	0.025	8	13.7	0.059
BSC	5	12	0	0.014						

Abbreviations: *OS* Overall survival, *LCR* Local control rate, *PFS* Progression free survival, *MST* Median survival time, *SCC* Squamous cell carcinoma antigen, *FIGO* International Federation of Gynecology and Obstetrics, *PORT* Post-operative radiation therapy, *serum SCC-Ag* Serum squamous cell carcinoma antigen, *serum CEA* Serum carcinoembryonic antigen, *CCRT* Concurrent chemoradiation therapy, *RT* Radiation therapy, *PALN* Para-aortic lymph nodes, *BSC* Best supportive care

**Fig. 3:**
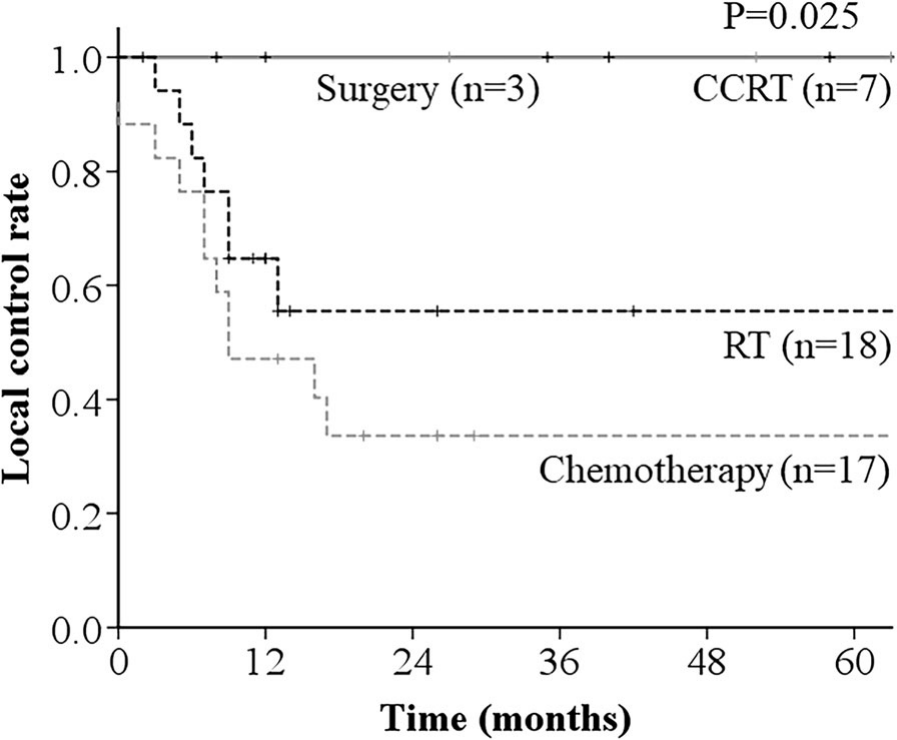
Local control rate (LCR) curves according to the recurrence treatment method. Patients treated with CCRT or surgery had 3-year LCRs of 100%, which were significantly superior to those of other patients (*p* = 0.025)

With respect to LCR according to the recurrence treatment method, the 3-year LCR was 100% for CCRT and surgery, 33.6% for chemotherapy, and 55.5% for RT (*p* = 0.025) (Fig. 3). The univariate analysis showed that the number of metastatic lesions (*p* = 0.001), serum SCC-Ag level (*p* = 0.012), and recurrence treatment method were significantly associated with LCR (Table 2).

**Fig. 4:**
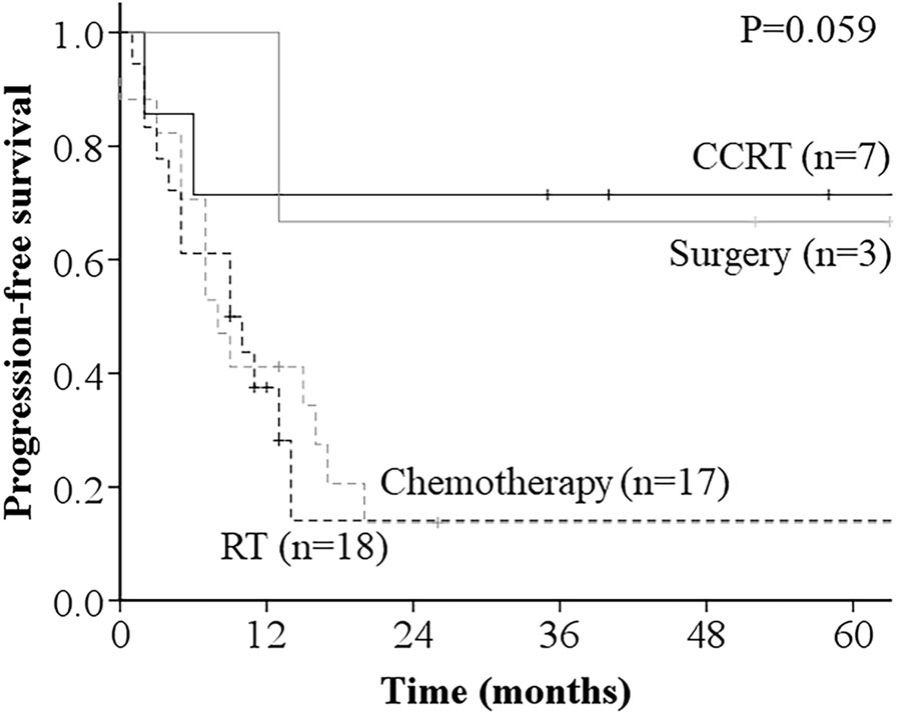
Progression free survival (PFS) curves according to the recurrence treatment method. Patients treated with CCRT or surgery presented superior PFS than other patients (*p* = 0.059)

With respect to PFS according to the recurrence treatment method, the 3-year PFS was 71.4% for CCRT, 66.7% for surgery, 13.7% for chemotherapy, and 14.1% for RT (*p* = 0.059) (Fig. 4). The univariate analysis showed that age at recurrence (*p* = 0.031), serum SCC-Ag (*p* = 0.002), and number of metastatic lesions (*p* = 0.009) were significantly associated with PFS (Table 2).

### Toxicity

No grade 3–5 acute gastrointestinal (GI) and genitourinary (GU) toxicities occurred in any group. Grade 3 late GU toxicity (ureteral stricture) occurred only in one patient, who had received CCRT. The ureteral stricture formed in both the initial and boost radiation fields. Hydronephrosis due to urethral stricture was treated using a urethral stent. In chemotherapy, surgery, and RT alone groups, no grade 3–5 GI and GU late toxicities were observed. There were no hematological grade 3/4 toxicities.

### Patterns of failure after RT or CCRT

A total of 15 patients experienced disease progression at 19 sites after RT or CCRT. Most of the failures (8/19) occurred in the supraclavicular or mediastinal lymph nodes. Two patients had recurrences in the pelvis, five patients had recurrences in PALNs, and four patients had other distant metastases. Among the patients with PALN recurrences, two patients had local recurrences and three patients had regional recurrences.

## Discussion

Salvage treatment modalities for isolated PALN recurrences after primary definitive cervical cancer treatment are equivocal. In the current study, we retrospectively examined a population of 50 patients with isolated PALN recurrence of cervical cancer. Our patient sample size was substantially larger than that of prior studies, and this is the first report to show the superiority of CCRT over other treatment modalities. Concurrent para-aortic irradiation and cisplatin-based chemotherapy generates an excellent prognosis, with a 3-year OS rate of 85.7%, 3-year LCR of 100%, and 3-year PFS of 71.4%. Previously, it has been suggested that RT confers long survival. Niibe et al. [5] reported on 84 patients who were treated with RT for isolated PALN recurrence after curative treatment, from 13 Japanese hospitals. Although the number of patients in the study seemed larger, the chemoradiotherapy group in the the study consisted mostly of sequential, non-CCRT. The 3- and 5-year overall survival rates of all patients were 49.5 and 31.3%, respectively. The authors indicated that irradiation of 51 Gy or greater was associated with better prognoses. Although 32 patients received chemo-radiation therapy in this study, a survival benefit of chemo-radiation therapy over RT alone was not identified. However, the chemo-radiation therapy in this study consisted mostly of sequential, nonconcurrent chemo-radiation therapy.

The reports from Singh et al. [7], Chou et al. [6], and Jeon et al. [8] support our findings. In a series of 14 patients, Singh et al. [7] demonstrated a 5-year OS of 100% in asymptomatic patients with isolated PALN who were treated with CCRT (*n* = 7), in which radiation doses ranged from 45 to 50.4 Gy. Chou et al. [6] examined 26 patients with isolated PALN recurrence after definitive RT, 14 of whom received CCRT, one received RT, and four received chemotherapy. They found that the 5-year survival rate of patients who received CCRT was 51.2%. Jeon et al. [8] examined 22 patients with PALN (*n* = 9), supraclavicular lymph node (*n* = 10), and inguinal lymph node (*n* = 3) recurrences. Patients who had both a long no-evidence-of-disease period and CCRT showed a 72.9% 5-year PFS rate and a 60% 5-year OS rate. In this study, concurrent chemotherapy was identified as an important factor for achieving a good prognosis. Radiation was delivered to PALN regions, up to 41.4–50.4 Gy, and then a radiation boost was administered to PALN recurrences, up to a total dose of 60 Gy. In our study, we found that patients who were treated with CCRT had better survival outcomes compared to those treated with other modalities. In patients treated with RT, RT was performed for the regional PALN area, up to a median dose of 44 Gy, followed by for the PALN metastases, up to a total median dose of 55 Gy. Regarding the total dose that should be administered to patients, considering results from previous studies [5–8] and the current study, we recommend administration of more than 50 Gy irradiation to PALN metastases.

With respect to the prognostic factors, serum SCC-Ag levels were significantly associated with LCR (*p* = 0.012) and PFS (*p* = 0.002). Jeon et al. [8] also reported that SCC-Ag levels above 8 ng/mL, at the time of recurrence, were associated with good prognoses. Contradictory to findings from other studies [7–9], our results demonstrated that the time to recurrence after initial therapy completion was not significantly associated with OS, LC, or PFS. Times to recurrence after initial therapy completion of more than 24 months, as reported by Singh et al. [7], and greater than 18 months, as reported by Jeon et al. [8], have been identified as good prognostic factors. Although we found that a median time to recurrence of more than 10 months tended to result in good prognosis, no significant differences were noted for even other cut-off durations, including 18 and 24 months. Kim et al. [9] reported on 12 patients with PALN recurrence after definitive or postoperative radiotherapy who were treated with concurrent chemotherapy and hyperfractionated RT with a total dose of 60 Gy in 50 fractions (1.2 Gy per fraction). They reported a 3-year survival rate of 19% and found more favorable outcomes in patients with a time to recurrence duration greater than 24 months after completion of initial therapy. Due to their inferior results, low dose per fraction may not be recommended.

Stereotactic body radiation therapy (SBRT) may provide a much higher radiation dose to the tumor with a steep dose gradient and reduce the radiation dose to normal tissues. Choi et al. [10] reported on 30 patients with isolated PALN metastases from uterine cervical and corpus cancer who were treated with SBRT (CyberKnife). The 4-year OS rate was 50.1%, and the 4-year LCR was 67.4% in their study, and they concluded that the SBRT results were promising. However, the LCR of CCRT observed in our study was not inferior to that of SBRT. Additionally, we recommend prophylactic regional PALN irradiation, especially for squamous cell carcinoma, because of difficulty in re-irradiation to re-recurrence in PALN. Only patients who are not candidates for long-course RT or have recurrences in previously irradiated fields may be candidates for SBRT.

Although the number of the patients was small in the current study, surgery also showed promise as a treatment, with an OS rate of 66.7% and LCR of 100%. Although surgical treatment is not widely accepted for patients such as ours, surgery has been reported to be feasible for PALN recurrences in colorectal cancer [11, 12]. For young patients and those with recurrences on the edge of the previous initial RT field, surgery might be a feasible treatment for PALN recurrences of cervical cancer. Debulking large nodes followed by PORT may be a good practice if the nodes are bulky and amenable to surgery.

On the basis of the reported results, multidisciplinary management might be a promising treatment for isolated PALN recurrences. If we consider the outcomes reported for the patients undergoing surgery as well as another treatment (most of them underwent adjuvant chemotherapy) and the outcomes for CCRT, there was a higher 3-year survival rate and better local control in the former group, while RT alone and chemotherapy alone was associated with a lower rate of survival (*p* = 0.036), local control (*p* = 0.006) and progression free survival (*p* = 0.007).

The study has some limitations. First, because this study was retrospective and performed at a single institution, careful consideration for possible selection bias is important. Clinical decisions for recurrent PALN treatment were frequently made based on time to recurrence after initial therapy completion, the location, number, and size of the metastatic lesions. For patients with a time to recurrence of less than 6 months and those with recurrence at the margin of the field of initial therapy, we often chose chemotherapy, rather than RT alone or CCRT. For patients with multiple and relatively larger metastatic lesions, we often chose CCRT, rather than RT alone. However, there were no significant differences between the treatment groups in terms of the assessed variables, except age at recurrence. Second, this study involved a relatively small number of patients. The clinical experience of fifty patients is insufficient to conclude that CCRT is superior to the other treatment modalities. However, isolated PALN recurrence is an infrequent occurrence after pelvic definitive therapy, and past studies have included a smaller number of patients. In the management of a relatively rare but curable oligo-recurrence, it is important to suggest this promising treatment strategy. Finally, the diagnoses of recurrent PALN were not histopathologically proven.

## Conclusions

In conclusion, although the patient cohort was small and there was selection bias, our results suggest that CCRT may effectively prevent local progression of disease recurrence in association with PALN and improve PFS and OS. Negative SCC-Ag and a small number of metastases may be one of the indicators of curable disease, and such patients may be candidates for salvage CCRT.

## Supplementary information

**Additional file 1: Table S1.** Patient characteristics of each treatment group.

## Data Availability

Not applicable.

## Abbreviations

BSC: Best supportive care
CCRT: Concurrent chemoradiation therapy
LCR: Local control rate
OS: Overall survival
PALN: Para-aortic lymph node
PFS: Progression free survival
PORT: Post-operative radiation therapy
RT: Radiation therapy
FIGO: Federation of Gynecology and Obstetrics
CT: Computed tomography
FDG-PET: [18F]-fluorodeoxyglucose positron emission tomography
SSC-Ag: Squamous cell carcinoma antigen
CEA: Carcinoembryonic antigen
IRB: Institutional Review Board
GI: Gastrointestinal toxicity
GU: Genitourinary toxicity
CTCAE: Common Terminology Criteria for Adverse Events
SBRT: Stereotactic body radiation therapy

## References

[1] Brady LW, Perez CA, Bedwinek JM (1986). Failure patterns in gynecologic cancer. Int J Radiat Oncol Biol Phys.

[2] Fagundes H, Perez CA, Grigsby PW, Lockett MA (1992). Distant metastases after irradiation alone in carcinoma of the uterine cervix. Int J Radiat Oncol Biol Phys.

[3] Carl UM, Bahnsen J, Rapp W (1992). Radiation therapy of Para-aortic lymph nodes in gynaecologic cancers: techniques, results and complications. Strahlenther Onkol.

[4] Grigsby PW, Vest ML, Perez CA (1994). Recurrent carcinoma of the cervix exclusively in the paraaortic nodes following radiation therapy. Int J Radiat Oncol Biol Phys.

[5] Niibe Y, Kenjo M, Kazumoto T, Michimoto K, Takayama M, Yamauchi C (2006). Multi-institutional study of radiation therapy for isolated Para-aortic lymph node recurrence in uterine cervical carcinoma: 84 subjects of a population of more than 5,000. Int J Radiat Oncol Biol Phys.

[6] Chou HH, Wang CC, Lai CH, Hong JH, Ng KK, Chang TC (2001). Isolated paraaortic lymph node recurrence after definitive irradiation for cervical carcinoma. Int J Radiat Oncol Biol Phys.

[7] Singh AK, Grigsby PW, Rader JS, Mutch DG, Powell MA (2005). Cervix carcinoma, concurrent chemoradiotherapy, and salvage of isolated paraaortic lymph node recurrence. Int J Radiat Oncol Biol Phys.

[8] Jeon W, Koh HK, Kim HJ, Wu HG, Kim JH, Chung HH (2012). Salvage radiotherapy for lymph node recurrence after radical surgery in cervical cancer. J Gynecol Oncol.

[9] Kim JS, Kim JS, Kim SY, Ki K, Cho MJ (2003). Hyperfractionated radiotherapy with concurrent chemotherapy for paraaortic lymph node recurrence in carcinoma of the cervix. Int J Radiat Oncol Biol Phys.

[10] Choi CW, Cho CK, Yoo SY, Kim MS, Yang KM, Yoo HJ (2009). Image-guided stereotactic body radiation therapy in patients with isolated Para-aortic lymph node metastases from uterine cervical and corpus cancer. Int J Radiat Oncol Biol Phys.

[11] Min BS, Kim NK, Sohn SK, Cho CH, Lee KY, Baik SH (2008). Isolated paraaortic lymph-node recurrence after the curative resection of colorectal carcinoma. J Surg Oncol.

[12] Choi PW, Kim HC, Kim AY, Jung SH, Yu CS, Kim JC (2010). Extensive lymphadenectomy in colorectal cancer with isolated Para-aortic lymph node metastasis below the level of renal vessels. J Surg Oncol.

