# Supplementary material for: Comparison of salvage therapies for isolated para-aortic lymph node recurrence in patients with uterine cervical cancer after definitive treatment
Source: Radiat Oncol. 2019 Dec 26;14:236. doi: 10.1186/s13014-019-1442-6 (PMC6933699; doi:10.1186/s13014-019-1442-6)
Supplement: Supplementary file 1 — Additional file 1: Table S1. Patient characteristics of each treatment group. [file 13014_2019_1442_MOESM1_ESM.docx]

| **Table S1.** Patient characteristics of each treatment group. | | | | | | | | | | |  |
| --- | --- | --- | --- | --- | --- | --- | --- | --- | --- | --- | --- |
|  | CCRT | | RT alone | | Chemo | | Surgery | | BSC | |  |
| Variable | No. | % | No. | % | No. | % | No. | % | No. | % | p |
| Histopathology |  |  |  |  |  |  |  |  |  |  |  |
| Squamous cell carcinoma | 6 | 86 | 16 | 89 | 12 | 71 | 3 | 100 | 3 | 60 |  |
| Adenocarcinoma | 1 | 14 | 1 | 6 | 4 | 24 | 0 | 0 | 1 | 20 |  |
| Adenosquamous carcinoma | 0 | 0 | 1 | 6 | 0 | 0 | 0 | 0 | 0 | 0 |  |
| Neuroendcrine carcinoma | 0 | 0 | 0 | 0 | 0 | 0 | 0 | 0 | 1 | 20 |  |
| Carcinoma with rhabdoid feature | 0 | 0 | 0 | 0 | 1 | 6 | 0 | 0 | 0 | 0 | 0.447 |
| Initial FIGO stage |  |  |  |  |  |  |  |  |  |  |  |
| Ⅰ | 1 | 14 | 4 | 22 | 6 | 35 | 0 | 0 | 0 | 0 |  |
| Ⅱ | 4 | 57 | 10 | 56 | 7 | 41 | 1 | 33 | 2 | 40 |  |
| Ⅲ | 2 | 29 | 3 | 17 | 3 | 18 | 1 | 33 | 1 | 20 |  |
| Ⅳ | 0 | 0 | 1 | 6 | 1 | 6 | 1 | 33 | 2 | 40 | 0.432 |
| Initial treatment |  |  |  |  |  |  |  |  |  |  |  |
| Surgery with PORT | 3 | 43 | 8 | 44 | 11 | 65 | 0 | 0 | 1 | 20 |  |
| Surgery without PORT | 1 | 14 | 3 | 17 | 1 | 6 | 0 | 0 | 0 | 0 |  |
| CCRT | 3 | 43 | 5 | 28 | 5 | 29 | 3 | 100 | 1 | 20 |  |
| RT alone | 0 | 0 | 2 | 11 | 0 | 0 | 0 | 0 | 3 | 60 | 0.066 |
| Age at the recurrence (year) |  |  |  |  |  |  |  |  |  |  |  |
| Median (range) | 51 (44-71) | | 64(26-84) | | 52(36-70) | | 54(44-56) | | 80(63-84) | | 0.008 |
| Performance status |  |  |  |  |  |  |  |  |  |  |  |
| 0-1 | 6 | 86 | 15 | 83 | 2 | 12 | 0 | 0 | 0 | 0 |  |
| 2-4 | 0 | 0 | 1 | 6 | 0 | 0 | 0 | 0 | 0 | 0 | 0.77 |
| Missing | 1 | 14 | 2 | 11 | 15 | 88 | 3 | 100 | 5 | 100 |  |
| Serum SCC at the recurrence |  |  |  |  |  |  |  |  |  |  |  |
| Positive | 3 | 43 | 11 | 61 | 10 | 59 | 0 | 0 | 2 | 40 |  |
| Negative | 3 | 43 | 6 | 33 | 3 | 18 | 3 | 100 | 2 | 40 | 0.156 |
| Missing | 1 | 14 | 1 | 6 | 4 | 24 | 0 | 0 | 1 | 20 |  |
| Serum CEA at the recurrence |  |  |  |  |  |  |  |  |  |  |  |
| Positive | 1 | 14 | 7 | 39 | 7 | 41 | 1 | 33 | 1 | 20 |  |
| Negative | 5 | 71 | 7 | 39 | 9 | 53 | 2 | 67 | 3 | 60 | 0.649 |
| Missing | 1 | 14 | 4 | 22 | 1 | 6 | 0 | 0 | 1 | 20 |  |
| Time interval between completion of initial treatment and PALN recurrence (month) |  |  |  |  |  |  |  |  |  |  |  |
| Median (range) | 6(1-66) | | 10(4-91) | | 15(1-57) | | 10(2-22) | | 17(1-31) | | 0.928 |
| Maximum size of PALN recurrence (mm) |  |  |  |  |  |  |  |  |  |  |  |
| Median (range) | 14(11-22) | | 18(9-26) | | 17(8-40) | | 11(10-11) | | 15(13-60) | | 0.115 |
| Number of recurrent PALN |  |  |  |  |  |  |  |  |  |  |  |
| 1–2 | 4 | 57 | 10 | 56 | 4 | 24 | 2 | 67 | 4 | 80 |  |
| ≥ 3 | 3 | 43 | 8 | 44 | 13 | 76 | 1 | 33 | 1 | 20 | 0.126 |

**Abbreviations:* FIGO= International Federation of Gynecology and Obstetrics; PORT= post-operative radiation therapy; serum SCC=serum squamous cell carcinoma antigen; serum CEA=serum carcinoembryonic antigen; CCRT= concurrent chemoradiation therapy; RT= radiation therapy; PALN= para-aortic lymph nodes; BSC= best supportive care
